# The influence of surface texture and wettability on initial bacterial adhesion on titanium and zirconium oxide dental implants

**DOI:** 10.1186/s40729-017-0093-3

**Published:** 2017-07-17

**Authors:** Torsten Wassmann, Stefan Kreis, Michael Behr, Ralf Buergers

**Affiliations:** 1 Present address: Department of Prosthodontics, University Medical Center Goettingen, Robert-Koch-Strasse 40, 37075 Goettingen, Germany; 2 Department of Prosthetic Dentistry, Regensburg University Medical Centre, Regensburg, Germany

**Keywords:** Zirconia, Titanium, Bacterial adhesion, Hydrophobicity, Roughness

## Abstract

**Background:**

This study aims to investigate bacterial adhesion on different titanium and ceramic implant surfaces, to correlate these findings with surface roughness and surface hydrophobicity, and to define the predominant factor for bacterial adhesion for each material.

**Methods:**

Zirconia and titanium specimens with different surface textures and wettability (5.0 mm in diameter, 1.0 mm in height) were prepared. Surface roughness was measured by perthometer (*R*_*a*_) and atomic force microscopy, and hydrophobicity according to contact angles by computerized image analysis. Bacterial suspensions of *Streptococcus sanguinis* and *Staphylococcus epidermidis* were incubated for 2 h at 37 °C with ten test specimens for each material group and quantified with fluorescence dye CytoX-Violet and an automated multi-detection reader.

**Results:**

Variations in surface roughness (*R*_*a*_) did not lead to any differences in adhering *S. epidermidis*, but higher *R*_*a*_ resulted in increased *S. sanguinis* adhesion. In contrast, higher bacterial adhesion was observed on hydrophobic surfaces than on hydrophilic surfaces for *S. epidermidis* but not for *S. sanguinis*. The potential to adhere *S. sanguinis* was significantly higher on ceramic surfaces than on titanium surfaces; no such preference could be found for *S. epidermidis*.

**Conclusions:**

Both surface roughness and wettability may influence the adhesion properties of bacteria on biomaterials; in this context, the predominant factor is dependent on the bacterial species. Wettability was the predominant factor for *S. epidermidis* and surface texture for *S. sanguinis*. Zirconia did not show any lower bacterial colonization potential than titanium. Arithmetical mean roughness values *R*_*a*_ (measured by stylus profilometer) are inadequate for describing surface roughness with regard to its potential influence on microbial adhesion.

## Background

Dental implants are one of the most frequently used treatment options for the replacement of missing teeth. The oral microflora and its dynamic interactions with the implant substrata seem to crucially influence the long-term success or failure of dental implants [1–6]. As soon as implant surfaces are exposed to the human oral cavity, they are immediately colonized by microorganisms [7, 8]. The initial bacterial adhesion on implants is the first and essential step in the geneses of complex peri-implant biofilms, which, in turn, may result in peri-implantitis and loss of the supporting bone [3].

The type of implant material and its specific texture and physico-chemical surface properties influence the quantity and quality of microbial colonization [1, 9–12]. In modern biomaterial research, implant surfaces are mainly modified to increase osseous integration into the alveolar bone; recently however, implant surfaces are also modified to reduce biofilm formation after exposure to the oral cavity. Innovative implant materials or surface modifications with reduced adhesion properties or even with antibacterial properties are of pertinent clinical interest [13, 14]. Up to now, monolithic titanium has been the most frequently used base material and gold standard for the construction of implant systems. Titanium is known for its excellent biocompatibility and outstanding mechanical properties [15]. Zirconia implant materials (ZrO_2_) were introduced as an alternative to titanium implants, mainly because of their supposedly reduced potential to adhere microorganisms [1, 16–19]. Surface roughness, texture, and wettability are regarded as the most significant surface factors influencing microbial accumulation on implants [9, 10, 12, 20]. Increased surface roughness on implant surfaces correlates with faster and firmer integration into the surrounding bone [21]. On the other hand, however, most studies indicate a positive correlation between surface roughness and the amount of adhering bacteria [1, 9–11, 19, 20, 22, 23]. For titanium implant surfaces, Bollen et al. found a threshold *R*_*a*_ value of 0.2 μm, and lower values did not further influence the quantity of bacterial adhesion [24]. In almost every corresponding investigation, the arithmetical mean roughness *R*_*a*_—which is measured by stylus profilometer—is used as a parameter to describe implant surface roughness. Rupp et al. showed that surfaces with very different morphologies may share the same *R*_*a*_ value. Furthermore, *R*_*a*_ values alone may be inadequate to describe “surface roughness” in respect to its potential influence on microbial adhesion [25]. For this reason, we additionally applied atomic force microscopy (AFM) for a three-dimensional assessment of the surface topography of the tested materials. AFM, which was developed to obtain fine details of a surface on a molecular scale, was found to be the most suitable instrument for surface roughness measurements [11, 26]. Furthermore, the crucial influence of surface wettability on bacterial adhesion is widely accepted, but there is still conflicting evidence if substrata with hydrophobic properties reduce or enhance the quantity of adhering microorganisms [9, 10, 27–31]. Although most studies describe surface roughness rather than wettability as the dominant factor for bacterial adhesion, the data on this matter is somewhat ambiguous [9–11, 20, 32–37]. So far, no study has yet varied surface roughness and hydrophobicity in well-defined patterns to define the crucial surface factor for different bacterial species.

The aim of the present in vitro study was to investigate bacterial adhesion (by means of the test species *Streptococcus sanguinis* and *Staphylococcus epidermidis*) on ten different titanium and zirconia implant surfaces. Surface texture and wettability were modified in well-defined patterns to correlate these surface properties with the amount of initially adhering bacteria and to define the predominant factor for each material and bacterial species.

## Methods

### Characterization of implant materials

In this study, we assessed two different implant materials in the form of round specimens (each measuring 5.0 mm in diameter and 1.0 mm in thickness, see Table 1). Half of the specimens were made of grade 1 pure titanium (Mechanische Werkstatt Biologie, University of Regensburg, Germany) and the other half of zirconia ceramic (IPS e.max ZirCAD; Ivoclar Vivadent, Ellwangen, Germany). The grade of the titanium used is the purest commercially available alloy. In comparison to other titanium grades, it is ductile and soft; however, there are very low amounts of impurities (≤1625%) and thus the lowest interferences caused by contained trace elements. The zirconia ceramic is a high-strength yttrium-stabilized zirconium oxide ceramic and as such a metal oxide ceramic. Due to its excellent mechanical properties, this ceramic is used in a wide range of indications.

**Table 1:**
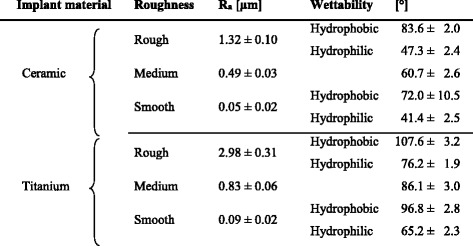
Arithmetic average of surface roughness *R*_*a*_ (means and standard deviations [μm]) and wettability (means and standard deviations [°]) of the ten tested material

Twenty specimens of each experimental implant material were subjected to one of the following surface treatments to modify surface roughness and surface free energy. The surface of some specimens was polished to high gloss with a polishing machine (Motopol 8; Buehler, Düsseldorf, Germany) and wet abrasive paper discs (Buehler, Lake Bluff, IL) with a grit of 1000, 2000, and 4000. Other specimens were sandblasted either with 50 or 250 μm aluminum trioxide at 2.5 bar for 20 s (both; Korox, Bego, Bremen, Germany). In the second part of the investigation, we additionally modified surface free energy values on the material surfaces of the rough and smooth substrata by applying n-propylsilane; hydrophilic conditions were altered by the application of aminosilane. As a result of various surface finishes (roughness and surface free energy) and the two starting materials (titanium and ceramic), there were finally ten different groups of test specimen with unique properties.

Surface roughness values of three specimens of each of the ten material groups were determined at three different sites with a stylus instrument (Perthometer S6P; Perthen, Göttingen, Germany) and shown as the arithmetic average peak-to-valley value (*R*_*a*_). Water contact angles (hydrophobicities) were calculated from automated contact angle measurements (OCA 15 plus; Dataphysics Instruments, Filderstadt, Germany) with deionized water. Nine drops of the liquid (one drop 1 μl) were examined on each substratum, and the contact angle was measured exactly 15 s after the positioning of the drop.

Three-dimensional images of rough and smooth implant surfaces were obtained by means of atomic force microscopy (AFM) using the tapping mode scan of an AFM VEECO machine (Plainview, USA); this method was also used to determine the surface topography. We scanned several randomly selected areas measuring either 3 μm × 3 μm or 30 μm × 30 μm for each of the test groups and sterilized all titanium specimens with UV light for 1 h before use.

### Microbial adhesion

We isolated a *S. epidermidis* strain culture (AC-Acession: AF270147) from the skin of one of the authors; the sample was identified and confirmed by 16S rDNA—nucleotide comparison (IDNS® version v3.1.63r14 © SmartGene 2005 Molecular Mycobacteriology). After isolation, *S. epidermidis* was proliferated in BHI—culture medium (Bacto™ Brain Heart Infusion, BD Becton, Dickinson and Company Sparks, MD, USA). Glycerine was added, and bacterial cultures were stored at −80 °C. Prior to testing, cultures were defrosted and incubated at 37 °C overnight. We cultivated *S. sanguinis* (strain 20068; DSMZ) in sterile trypticase soy broth (Tryptic Soy Broth; BD Diagnostics, Sparks, MD, USA) supplemented with yeast extract (Sigma-Aldrich, St. Louis, Mo, USA). For both types of bacteria, cells were harvested by centrifugation, washed twice in phosphate-buffered saline (PBS) (Sigma-Aldrich, St. Louis, Mo, USA), and resuspended in normal saline. After that, we adjusted the cells by densitometry (Genesys 10S; Thermo Spectronic, Rochester, NY, USA) at 600 nm to a MacFarland 0.4 standard optical density that equalled the bacterial concentration of approximately 5 × 10 ^9^ cfu (colony forming units)/ml.

We determined the quantity of bacterial adhesion with a fluorescence dye, i.e., the CytoX-Violet Cell Proliferation Kit (Epigentek Group Inc., New York, USA), and recorded fluorescence intensities with an automated multi-detection reader (Fluostar optima; BMG labtech, Offenburg, Germany) at wavelengths of 560 nm excitation and 590 nm emission. High relative fluorescence intensities indicate high numbers of viable adhering bacteria. For simulating the influence of a salivary pellicle, we incubated specimens in 48-well plates with 1 ml of artificial saliva for 2 h prior to adhesion testing [2]. We then removed the saliva, added 1 ml of bacterial suspension to each well, and incubated the well plates at 37 °C for 120 min on an orbital shaker. After biofilm formation, we extracted the bacterial solution by suction and washed the specimens once with PBS to remove non-adherent bacteria. All specimens were transferred to a new 48-well plate. For each well, we added 200 μl PBS and 20 μl CytoX-Violet (indicator solution) and incubated the well plates at 37 °C for 120 min in darkness; 190 μl of the indicator solution from each well was transferred to sterile black 96-well plates, and fluorescence intensities were recorded.

Ten specimens of each material group tested were investigated. As control references, we used the fluorescence values of pure phosphate-buffered saline (0-control), buffer and CytoX-Violet (dye-control), and pure bacterial solution (bacteria-control).

### Statistical analysis

All calculations and graphic displays were done with SPSS 16.0 for Windows (SPSS Corporation, Chicago, IL, USA). Means and standard deviations for *R*_*a*_, water contact angles, and relative fluorescence intensities were calculated. We used three-way analysis of variance (ANOVA) to analyze the influence of *R*_*a*_ and hydrophobicity on the adherence of *S. sanguinis* and *S. epidermidis* to the titanium and ceramic specimens. The Tukey–Kramer multiple comparison test was applied for post hoc analysis, and the level of significance was set at *α* = 0.05.

## Results

### Characterization of implant material groups

The median surface roughness values (*R*_*a*_) of each material group (*n* = 10) tested are shown in Table 1. The differences in *R*_*a*_ between rough, medium, and smooth specimens were statistically significant for ceramic as well as for titanium (*p* < 0.01 for all comparisons). The roughness values of rough and medium ceramic specimens (1.32 μm/0.49 μm) were significantly lower than those of titanium specimens (2.98 μm/0.83 μm; *p* < 0.01 for both comparisons). No significant difference was found between the *R*_*a*_ of smooth titanium and smooth ceramic specimens (0.09 μm/0.05 μm; *p* = 0.983).

The median water contact angles (wettability) of each specimen are given in Table 1. All four hydrophobic surfaces showed significantly higher contact angles than the corresponding hydrophilic surfaces (*p* < 0.01 for rough ceramic, smooth ceramic, rough titanium, and smooth titanium). Roughness values did not change after hydrophilization or hydrophobization (data not shown).

**Fig. 1:**
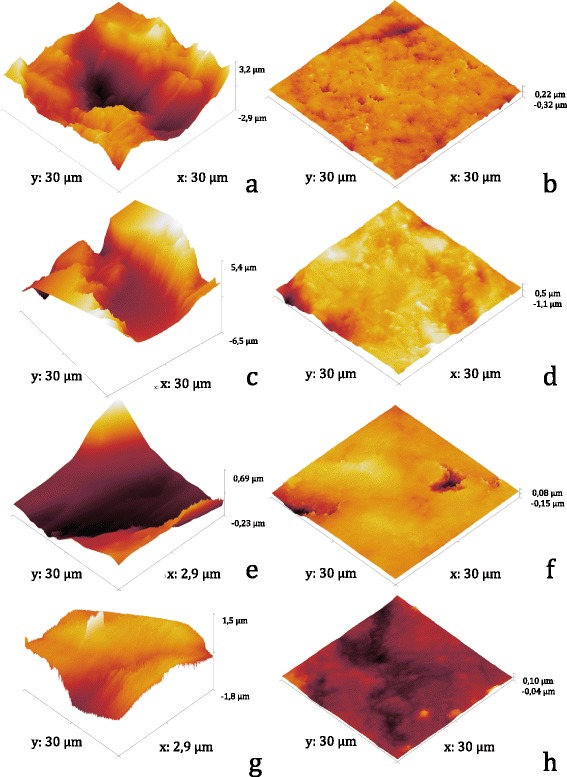
AFM images for 30 μm × 30 μm (**a**–**d**) and 3 μm × 3 μm scan areas (**e**–**h**) of rough ceramic (**a**, **e**), smooth ceramic (**b**, **f**), rough titanium (**c**, **g**), and smooth titanium (**d**, **h**)

**Fig. 2:**
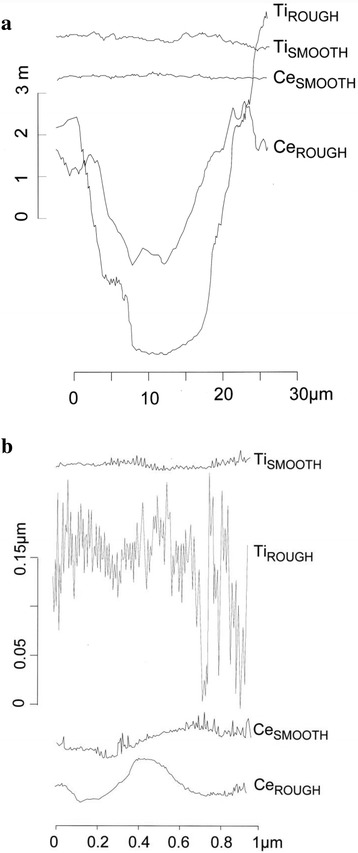
Comparison of AFM surface profiles of rough ceramic (Ce_ROUGH_), smooth ceramic (Ce_SMOOTH_), rough titanium (Ti_ROUGH_), and smooth titanium (Ti_SMOOTH_); scan sizes are 30 μm in **a** and 1 μm in **b**

Examples of the atomic force micrographs are given in Fig. 1a–d (30 μm × 30 μm = 900 μm^2^ scan area), e–h (3 μm × 3 μm = 9 μm^2^ scan area). Considerably higher roughness values could be observed on the sandblasted ceramic and titanium surfaces than on the corresponding polished surfaces. Neither the 900 μm^2^ scan areas nor the corresponding AFM roughness profiles showed any well-defined differences between ceramic and titanium for smooth and rough specimens (Fig. 2a). On closer examination (9 μm^2^ scan areas), small grooves (measuring approximately 0.5 μm in diameter and 0.08 μm in height) could be observed on the smooth ceramic substrata (Fig. 1g), whereas the smooth titanium surfaces seemed to be totally plane (Fig. 1h). Furthermore, the microstructure of rough titanium appeared to be significantly more irregular than the smooth titanium surface and both ceramic surfaces (Fig. 2b).

### Influence of surface roughness on bacterial adhesion

**Fig. 3:**
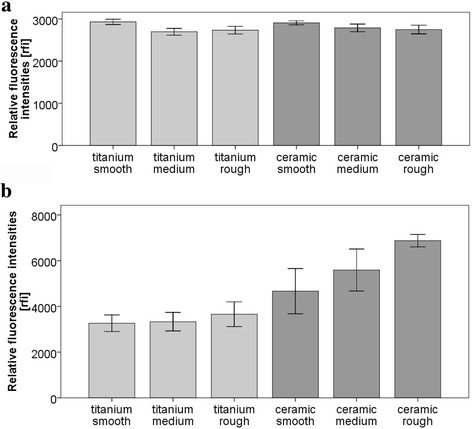
Relative fluorescence intensities (rfi) of *S. epidermidis* (**a**) and *S. sanguinis* (**b**) on titanium and ceramic implant surfaces with different grades of roughness (means and standard deviations)

The relative fluorescence intensities (rfi) for *S. epidermidis*, indicating the quantity of adhering staphylococci, narrowly varied between 2931 and 2697 relative fluorescence units (rfu) (Fig. 3a). Except for smooth titanium (2931 ± 99 rfu), on which significantly more adhering bacteria were found than on medium titanium (2697 ± 127 rfu; *p* = 0.002) and rough titanium (2734 ± 145 rfu; *p* = 0.014), variations in surface roughness did not lead to any differences in adhering *S. epidermidis*. The differences in staphylococcal adhesion on smooth (2908 ± 74 rfu), medium (2789 ± 143 rfu), and rough (2749 ± 162 rfu) ceramic specimens were not statistically significant (*p* > 0.05 for all comparisons).

In general, significantly more *S. sanguinis* adhered to ceramic surfaces than to titanium surfaces (*p* < 0.05 for all comparisons, except for smooth ceramic compared with rough titanium: *p* = 0.244) (Fig. 3b). Titanium specimens (smooth titanium 3263 ± 475 rfu; medium titanium 3331 ± 641 rfu; rough titanium 3656 ± 855 rfu) tended to show higher streptococcal adhesion on rough surfaces in comparison to medium and smooth surfaces, but the differences between the tested material groups were not statistically significant (*p* > 0.05 for all comparisons). On ceramic surfaces (smooth ceramic 4668 ± 1562 rfu; medium ceramic 5590 ± 1493 rfu, rough ceramic 6875 ± 428 rfu), higher surface roughness led to increased *S. sanguinis* adhesion (*p* < 0.05 for all comparisons, except for smooth ceramic compared with medium ceramic: *p* = 0.244).

### Influence of surface wettability (hydrophobicity) on bacterial adhesion

**Fig. 4:**
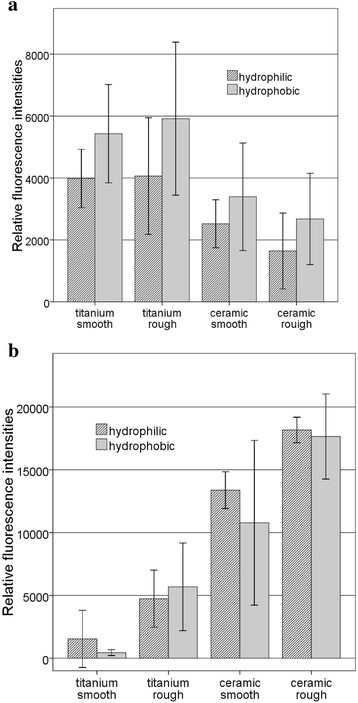
Relative fluorescence intensities (rfi) of *S. epidermidis* (**a**) and *S. sanguinis* (**b**) on titanium and ceramic implant surfaces with different grades of roughness and hydrophobicity (means and standard deviations)

*S. epidermidis* (Fig. 4a) tended to show higher bacterial adhesion on hydrophobic surfaces (titanium smooth 5337 ± 1511 rfu, titanium rough 5916 ± 2472 rfu, ceramic smooth 3395 ± 1738 rfu, and ceramic rough 2676 ± 1476 rfu) than on hydrophilic surfaces (titanium smooth 3897 ± 985 rfu, titanium rough 5662 ± 1884 rfu, ceramic smooth 2522 ± 775 rfu, and ceramic rough 1644 ± 1225 rfu), but these differences were not statistically significant (*p* > 0.05 for all comparisons). A comparison of rough and smooth specimens did not show any differences in staphylococcal adhesion (*p* > 0.05 for all comparisons).

In general, the potential to adhere *S. sanguinis* was significantly higher for all ceramic surfaces—hydrophobic and hydrophilic—than for titanium specimens (*p* < 0.05 for all 16 comparisons) (Fig. 4b). A comparison of hydrophobic and hydrophilic surfaces did not show any statistically significant differences (for smooth titanium: *p* = 0.997; for rough titanium: *p* = 0.999; for smooth ceramic: *p* = 0.723; and for rough ceramic: *p* > 0.999). Hydrophilic titanium and hydrophilic ceramic surfaces did not show any statistically significant differences between rough and smooth surfaces (*p* > 0.05 for both comparisons).

## Discussion

The problems involved in osseous healing of dental implants appear to be largely solved. Biofilm formation on exposed implant and abutment surfaces, however, is a fortiori crucial for the long-term therapeutic success of an implant, because biofilms are the most frequent cause of peri-implantitis and implant loss [3–7]. Consequently, new implant surface modifications with reduced properties to accumulate microorganisms or even with antibacterial properties are of pertinent clinical interest [8, 9]. In general, the physico-chemical surface properties of an implant—influenced by the type of material, its surface morphology, and surface coatings—define the potential to adhere oral microorganisms [4, 10, 11]. In this context, surface roughness and hydrophobicity seem to be the main material-linked factors influencing microbial adhesion and biofilm formation on implant surfaces [12, 13]. Therefore, the main object of the present study was to investigate bacterial adhesion on different titanium and ceramic implant surfaces, to correlate these findings with surface roughness and surface hydrophobicity, and to define the predominant factor for bacterial adhesion for each material group.

### Implant materials and biological potentials

In dental implantology, titanium is the most frequently and most successfully used “gold standard” material because of its biocompatibility and excellent mechanical properties. The surface structure of titanium can be modified very easily by sandblasting, acid etching, plasma spraying, etc. to optimize integration into the surrounding bone [14]. Recently, high-strength zirconia implant materials (ZrO_2_) have been invented as an alternative to titanium because of their resistance to corrosion and their enhanced esthetics in case of exposure and because dental ceramics are generally regarded as biomaterial with low potential to accumulate biofilms [15–18]. In fact, very little information is available on the microbial performance of zirconium implant materials. Some recent studies about biofilm formation on implant surfaces have concluded that zirconium oxide may have lower bacterial colonization potential than titanium [4, 18], an effect that is attributed to the specific chemical structure and the resulting electric conductivity of zirconia [4, 10, 19]. In contrast, other studies have not indicated such superiority of zirconia with regard to its microbial performance but have shown that the development of biofilm is not influenced by the type of material surface [9, 10, 20, 21]. The results of the present study are not unambiguous with regard to the influence of the substratum material (titanium vs. zirconia) on bacterial adhesion. We could not find any difference between the bacterial accumulation on titanium and ceramic for *S. epidermidis*, but the potential to adhere *S. sanguinis* was significantly higher on ceramic than on titanium. Some authors reported antibacterial effects for titanium, which may be a further explanation for the rather low amounts of adhering bacteria on titanium [22, 23]. Furthermore, titanium is coated by a layer of surface oxide, which physical and mechanical characteristics are more closely related to ceramic than to metal. This phenomenon may explain why similar protein-binding properties on titanium and zirconium oxide have been reported and why zirconia did not show any reduced bacterial adhesion in the present study [20].

### Surface roughness and shear forces

Besides, the surface material itself and its chemical composition, surface roughness, and hydrophobicity have a crucial influence on the accumulation of microorganisms. In most previous studies on bacterial adhesion on titanium and ceramic surfaces, the quantity of bacterial adhesion showed a direct positive correlation with surface roughness [4, 10, 18, 24–26]. In case of interacting surface roughness and hydrophobicity, roughness seems to be dominant in in vitro settings [11, 25, 27]. This phenomenon is enhanced in vivo because of the sheltering effect of rough surfaces against the removal forces present in the oral cavity [10, 28–30]. These observations were confirmed by one of our own studies, in which in vivo and in vitro initial bacterial adhesion followed the circular surface irregularities, consisting of the grinding tracks generated by the machine manufacturing of the specimen with a lathe [25]. Nevertheless, two in vivo studies reported contradictory observations on the impact of surface roughness on bacterial adhesion. Gatewood et al. [31] and Wennerberg et al. [32] worked with volunteers who carried specimens in their periodontal pockets respectively modified implant abutments for a test period up to 4 weeks and could not find any different amounts of adhering oral biofilms, neither on rough nor on smooth titanium surfaces.

In most in vivo studies on this matter, specimens are mounted on individual splints and thus exposed to shear forces related to salivary flow, muscles, and chewing activity [4, 10, 25, 33]. With regard to the “real in situ situation,” no corresponding removal forces are present in the peri-implant region, which is protected from such forces by the adjacent peri-implant mucosa. The tight contact between the peri-implant soft tissues and the implant abutment surface protects implant surfaces from extensive shear forces. Therefore, shear forces and the influence of surface roughness may be overestimated in these specific settings. As a result, we choose a semi-static experimental setup, in which specimens were placed in an orbital shaker to simulate fluid movements in the peri-implant sulcus. This consideration was approved by the findings of Elter et al. who investigated supra- and subgingival biofilm formation on implant abutments with different roughness values. Biofilm accumulation in supragingival areas was shown to be significantly increased by higher *R*_*a*_ values, whereas this correlation was not found in subgingival areas [5].

In the present study, sandblasting (with 50 or 250 μm aluminum trioxide) resulted in significant increases of *R*_*a*_ on titanium and ceramic surfaces. These *R*_*a*_ values were higher than those for commercially available implant abutments (observed to range from 0.10 to 0.30 μm) [35]. According to the classification by Albrektsson and Wennerberg, smooth ceramic and titanium materials and the medium ceramic material were classified as “smooth” (*R*_*a*_ < 0.5 μm), the medium titanium material as “minimally rough” (*R*_*a*_ 0.5–1.0 μm), the rough ceramic material as “moderately rough” (*R*_*a*_ 1.1–2.0 μm), and the rough titanium material as “rough” (*R*_*a*_ > 2.0 μm) [36]. Although titanium and zirconia had the same treatment, polishing and sandblasting resulted in significantly higher *R*_*a*_ values on the titanium specimens than on the zirconia specimens. For titanium, Bollen et al. and Quirynen et al. evaluated a threshold *R*_*a*_ of 0.2 μm; below this threshold, a change in roughness did not significantly affect the quantity of plaque accumulation [27, 37]. The medium and rough surfaces in the present study showed *R*_*a*_ values above the threshold of 0.2 μm; therefore, a correlation between *R*_*a*_ and bacterial adhesion should be expected. Surprisingly, in the present study, surface roughness (*R*_*a*_) did not influence the quantity of adhering *S. epidermidis*, neither on titanium nor on zirconia. For *S. sanguinis*, such correlation was observed for zirconia but not for titanium. A possible explanation for this phenomenon can be found in the AFM observations. On closer examination (9 μm^2^ scan areas, see Fig. 2b) and from a bacterial point of view (a single cell measures approximately 1 μm in diameter), no significant differences in surface profile or morphology could be found between all surfaces tested (except for rough titanium). From a microscopic or an AFM viewpoint, most surfaces are rough no matter how fine the finish; therefore, all types of surfaces provide adequate adhesion conditions for microbial accumulation [1]. The large-scale surface irregularities (>30 μm) on the sandblasted titanium and zirconia specimens, which were observed during the examination of the 900 μm^2^ scan areas (Fig. 2a) and which were indicated by high *R*_*a*_ values, did not influence bacterial adhesion in the present semi-static experimental setup. However, these irregularities will probably increase microbial adhesion in an in vivo testing with supragingival exposition of specimens, when the influence of intraoral shear forces becomes apparent [25, 28, 29]. In contrast, the small grooves (measuring approximately 0.5 μm in diameter) on smooth zirconia surfaces in AFM may possibly explain the enhanced potential to adhere bacteria in contrast to totally plane titanium surfaces, because initial microbial colonization has been shown to start from very small—and not from large-scale—pits and gullies [25, 26, 38, 39]. In summary, characterizing the influence of surface morphology on initial bacterial adhesion (in the semi-static and static environment such as the peri-implant) by surface roughness values such as *R*_*a*_ alone is rather inadequate because of the requirement of an additional three-dimensional analysis of the microstructure. These observations were confirmed by Barbour et al. who observed different bacterial coverage on surfaces of the same roughness but different detailed surface morphology [40]. The different adhesion properties of *S. epidermidis* and *S. sanguinis* with regard to the influence of surface morphology may result from morphologic differences between the bacterial species. Accordingly, Barbour et al. observed that *Actinomyces naeslundi* adhere better to smooth surfaces than to rough surfaces, whereas *Streptococcus mutans* prefer rough substrata [40]. In addition, Taylor et al. could not clearly relate surface roughness of PMMA surfaces to the amount of adhering *S. epidermidis*, which supports the results of the present study [41].

### Surface free energy and hydrophobicity

Besides surface roughness and morphology, the hydrophobicity and surface free energy (SFE) of an implant surface are known to influence bacterial adhesion [42, 43]. Physico-chemical interactions (non-specific) are composed of van der Waals forces, electrostatic interactions, and acid-based interactions, which in turn define the surface free energy of a substratum [44]. The surface free energy can be calculated by contact angle measurement of different liquids with differing hydrophobicities [25] or by measuring the wettability by determining water contact angles [45]. Results from different studies that relate surface free energy and hydrophobicity to microbial adhesion are conflicting [44, 46]. However, it has become apparent that, according to the thermodynamic model of microbial adhesion, hydrophobic materials are preferentially colonized by hydrophobic bacteria and vice versa [39, 44, 47–49]. Consequently, the adhesion properties of different bacteria are affected by the hydrophobicity of the bacterial cell surface [11, 44]. Both *S. epidermidis* and *S. sanguinis* are known to be rather hydrophobic; therefore, hydrophobic surfaces are preferable [44, 49]. Accordingly, Drake et al. reported that titanium samples with hydrophobic surfaces have higher levels of bacterial colonization of *S. sanguinis* than titanium samples with hydrophilic surfaces [50]. Surface roughness itself is known to influence hydrophobicity [51], but many studies have also clearly shown that minor variations in surface roughness do not significantly affect hydrophobicity values [12]. In the present study, different specimens with varying hydrophobicity but similar surface roughness were selected to eliminate the influence of surface roughness. To our knowledge, this is the first in vitro study to vary surface roughness and hydrophobicity in well-defined patterns to define the predominant factor for the two single-species biofilms tested. For *S. sanguinis*, no significant difference could be found with regard to bacterial adhesion between the hydrophobic and hydrophilic surfaces of zirconia and titanium. In contrast, *S. epidermidis* showed higher initial adhesion on hydrophobic than on hydrophilic surfaces; this finding can be attributed to the hydrophobic properties of *S. epidermidis* and explained by the thermodynamic model of microbial adhesion.

### Biofilm models

In vivo biofilm models with multi-species biofilms offer the opportunity to evaluate materials in simulated clinical conditions including composite plaque, salivary pellicle, and removal forces [18]. Although the understanding of oral biofilms and the influence of surface characteristics on microbial accumulation has increased, significant gaps in the fundamental knowledge about the formation and establishment of such microbial communities still exist. Furthermore, the most essential processes in oral biofilm formation are not yet fully understood [52]. Therefore, it is necessary to examine the correlation between bacterial adhesion—including differences between different species—and modifications of surface characteristics in simplified, reproducible, and manageable in vitro systems to transfer the knowledge on fundamental in vitro matters to new clinical biomaterial implementations. Additionally, we indicated in a previous study the possibility of a correlation between in vivo and semi-static in vitro findings in respect to microbial adhesion on surfaces with different surface properties [25]. Even in a simplified in vitro setting, the quantity and quality of bacterial accumulation are influenced by many factors; in vitro relationships between surface characteristics and bacterial adhesion depend on experimental conditions, such as preconditioning protein films and the simulation of shear forces [8, 53]. For example, salivary proteins mediate the initial accumulation of microorganisms in the human oral cavity [54]. For simulating the influence of the salivary pellicle in vitro, specimens may be incubated in various saliva solutions before bacterial adhesion testing. In the present study, all specimens were pre-incubated with artificial saliva [2], which was chosen to exclude the influence of inter-individual variations in salivary protein content and the composition of human saliva so that reproducible results could be achieved [26, 55]. Two different single-species biofilms, *S. epidermidis* and *S. sanguinis*, were used as test microorganisms to investigate the potential of differently treated implant surfaces to adhere these bacteria. *S. epidermidis* and *S. sanguinis* are not usually associated with active peri-implantitis, but they are amongst the main early colonizers of oral tissues and artificial biomaterials, paving the way for more pathogenic species [56–58]. *S. epidermidis* and *S. sanguinis* represent two dominant but very different bacterial families, i.e., Streptococcaceae and Staphylococcaceae, which are members of the human oral microbiome; these bacteria normally reside on the mucous membranes of humans and can bind to hard surfaces in the oral cavity [57]. *S. sanguinuis* is commonly present in the human oral cavity and known as a pioneer bacterium of oral biofilms [10, 18, 56, 58, 59]. *S. epidermidis*, normally a commensal bacterium of the skin, is a major concern for patients with surgical implants, causing the growth of pathogenic biofilms on various implant devices, such as breast and hip implants, which may result in implant failure [60]. In some recent studies, *S. epidermidis* has also been detected in pathogenic biofilms on failing dental implants [43]. Fluorometric techniques offer the opportunity to quantitatively investigate a high number of specimens in a short period of time and, at the same time, provide reproducible and significant data [25]. In this study, the CytoX-Violet Cell Proliferation Assay Kit was used to simply measure the amount of viable bacteria adhering to the test specimens. The fluorometric change of the indicator solution shows the activity of the cellular dehydrogenases and is directly proportional to the cell viability of adhering bacteria. It should be mentioned that this specific method fails to indicate vital adhering bacteria and cannot differentiate between cultivable vital cells and non-cultivable vital cells. This is important because large amounts of dead bacteria (up to 40%) have already been found after short incubation times [25].

## Conclusions

Within the limitations of an in vitro study, our results indicate that surface roughness as well as wettability may influence the adhesion properties of bacteria on implant surfaces. Furthermore, the predominant factor for adhesion depends on the bacterial species itself. Zirconia implant material did not show any lower bacterial colonization potential than titanium. The influence of substratum material, surface texture, and wettability of implant surfaces on microbial adhesion does not exactly follow universal rules and differs between bacterial species. Additionally, arithmetical mean roughness values *R*_*a*_ (measured by stylus profilometer) are inadequate for describing surface roughness in respect to its potential influence on microbial adhesion. Future studies may use more sophisticated techniques such as confocal microscopy, wide-angle confocal microscopy or laser scanning microscopy in order to gain precise three-dimensional topographical values and to evaluate their influence on microbial adhesion.
